# The genome sequence of the orange-tip butterfly, *Anthocharis cardamines* (Linnaeus, 1758)

**DOI:** 10.12688/wellcomeopenres.18117.1

**Published:** 2022-10-13

**Authors:** Sam Ebdon, Gertjan Bisschop, Konrad Lohse, Ilik Saccheri, James Davies, Wellcome Sanger Institute Tree of Life programme, Wellcome Sanger Institute Scientific Operations: DNA Pipelines collective, Tree of Life Core Informatics collective, Darwin Tree of Life Consortium

**Affiliations:** 1 Institute of Evolutionary Biology, University of Edinburgh, Edinburgh, UK; 2 Department of Evolution, Ecology and Behaviour, University of Liverpool, Liverpool, UK

**Keywords:** Anthocharis cardamines, orange-tip, genome sequence, chromosomal, Lepidoptera

## Abstract

We present a genome assembly from an individual female *Anthocharis cardamines* (the orange-tip; Arthropoda; Insecta; Lepidoptera; Pieridae). The genome sequence is 360 megabases in span. The majority (99.74%) of the assembly is scaffolded into 31 chromosomal pseudomolecules, with the W and Z sex chromosomes assembled. Gene annotation of this assembly on Ensembl has identified 12,477 protein coding genes.

## Species taxonomy

Eukaryota; Metazoa; Ecdysozoa; Arthropoda; Hexapoda; Insecta; Pterygota; Neoptera; Endopterygota; Lepidoptera; Glossata; Ditrysia; Papilionoidea; Pieridae; Pierinae; *Anthocharis*; *Anthocharis cardamines* (Linnaeus, 1758) (NCBI:txid227532).

### Background

The orange-tip butterfly ( *Anthocharis cardamines*) is a member of the Anthocharidini, a tribe within the Pierinae ( Wahlberg *et al.,* 2014), with a Palearctic distribution, including throughout the British Isles, where two subspecies are recognised, *britannica* (mainland Britain) and *hibernica* (Ireland and Isle of Man). The English population exhibits a reduced number of chromosomes (n = 30) compared to specimens from continental Europe (n = 31), implying a fusion event since the separation of England from the continent ~7,000 years ago ( Bigger, 1978). Following range contraction on the British mainland in the late 19 ^th^ century, which left disjunct populations in England and Northeast Scotland, the species began recolonizing these regions in the mid-20 ^th^ century ( Long, 1979), and has shown an increasing trend to the present ( Fox *et al.,* 2015). *A. cardamines* is listed as Least Concern in the IUCN Red List (Europe) ( van Swaay *et al.,* 2010). The spring flight period is phenologically responsive to temperature and climate change ( Prieto & Destouni, 2015). The species is polyphagous on Brassicaceae, usually *Cardamine pratensis* and *Alliaria petiolata* in Britain ( Courtney & Duggan, 1983), inhabiting flowery meadows, woodland borders, riverbanks, hedgerows and gardens. Host-plant use influences adult emergence schedule, size and dispersal behaviour ( Davies & Saccheri, 2013).

### Genome sequence report

**Figure 1.**
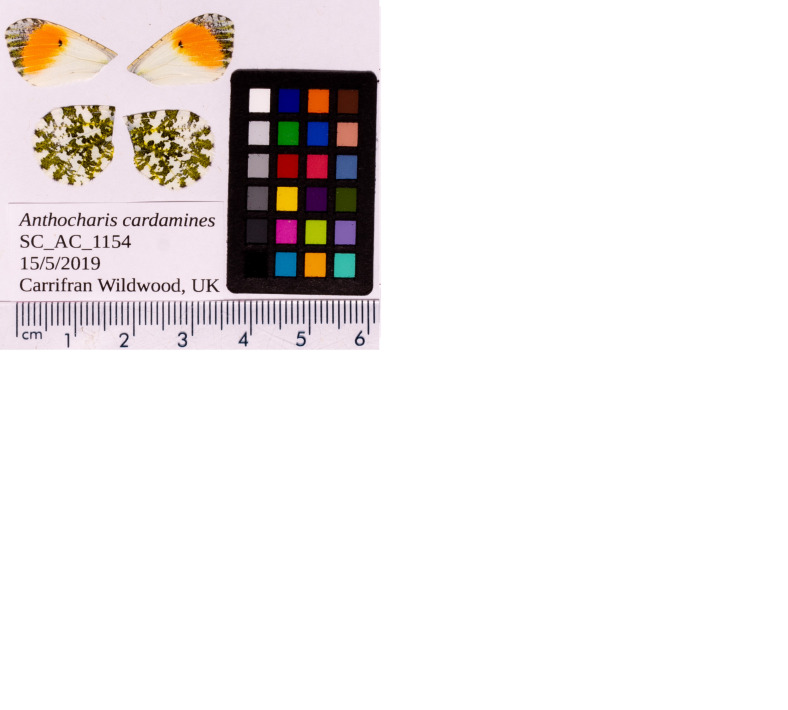
Forewings and hindwings of the *Anthocharis cardamines* specimen from which the genome was sequenced. Dorsal (top left) and ventral (top right) surface view of wings from specimen SC_AC_1156 (ilAntCard3) and dorsal (bottom left) and ventral (bottom right) surface view of wings from specimen SC_AC_1154 (ilAntCard2) from Scotland, UK. ilAntCard3 was used to generate Pacific Biosciences and 10X genomics data and ilAntCard2 was used to generate Hi-C data.

The genome was sequenced from a single female *A. cardamines* ( Figure 1) collected from Carrifran Wildwood, Scotland (latitude 55.4001, longitude -3.3352). A total of 69-fold coverage in Pacific Biosciences single-molecule circular consensus (HiFi) long reads and 97-fold coverage in 10X Genomics read clouds were generated. Primary assembly contigs were scaffolded with chromosome conformation Hi-C data. Manual assembly curation corrected 50 missing/misjoins and removed 2 haplotypic duplications, reducing the assembly length by 0.53% and the scaffold number by 40.00%, and increased the scaffold N50 by 5.62%.

**Figure 2.**
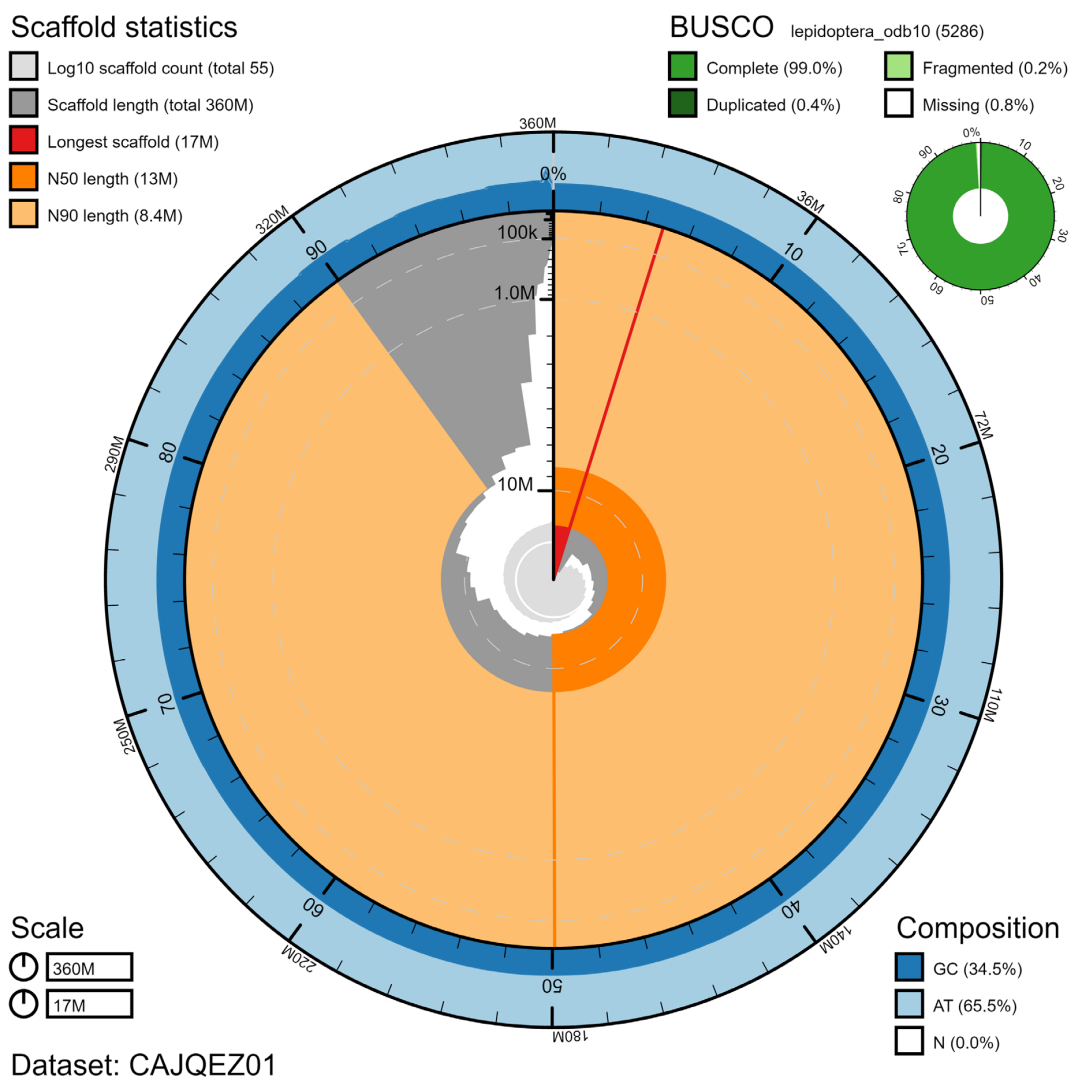
Genome assembly of *Anthocharis cardamines*, ilAntCard3.1: metrics. The BlobToolKit Snailplot shows N50 metrics and BUSCO gene completeness. The main plot is divided into 1,000 size-ordered bins around the circumference with each bin representing 0.1% of the 359,616,706 bp assembly. The distribution of chromosome lengths is shown in dark grey with the plot radius scaled to the longest chromosome present in the assembly (17,367,467 bp, shown in red). Orange and pale-orange arcs show the N50 and N90 chromosome lengths (12,507,586 and 8,384,805 bp), respectively. The pale grey spiral shows the cumulative chromosome count on a log scale with white scale lines showing successive orders of magnitude. The blue and pale-blue area around the outside of the plot shows the distribution of GC, AT and N percentages in the same bins as the inner plot. A summary of complete, fragmented, duplicated and missing BUSCO genes in the lepidoptera_odb10 set is shown in the top right. An interactive version of this figure is available at https://blobtoolkit.genomehubs.org/view/ilAntCard3.1/dataset/CAJQEZ01/snail.

The final assembly has a total length of 360 Mb in 54 sequence scaffolds with a scaffold N50 of 12.5 Mb ( Table 1). The majority, 99.74%, of assembly sequence was assigned to 31 chromosomal-level scaffolds, representing 29 autosomes (numbered by sequence length), and the W and Z sex chromosome ( Figure 2– Figure 5; Table 2). The assembly has a BUSCO v5.1.2 ( Manni *et al.,* 2021) completeness of 99.0% (single 98.6%, duplicated 0.4%) using the lepidoptera_odb10 reference set (n=5,286). While not fully phased, the assembly deposited is of one haplotype. Contigs corresponding to the second haplotype have also been deposited.

**Table 1.** Genome data for *Anthocharis cardamines*, ilAntCard3.1.

*Project accession data*	
Assembly identifier	ilAntCard3.1
Species	*Anthocharis cardamines*
Specimen	ilAntCard3 (genome assembly); ilAntCard2 (Hi-C)
NCBI taxonomy ID	NCBI:txid227532
BioProject	PRJEB43792
BioSample ID	SAMEA7523110
Isolate information	Female, abdomen (ilAntCard3); male, whole organism (ilAntCard2)
*Raw data accessions*	
PacificBiosciences SEQUEL II	ERR6544655
10X Genomics Illumina	ERR6054580-ERR6054583
Hi-C Illumina	ERR6054584
*Genome assembly*	
Assembly accession	GCA_905404175.1
*Accession of alternate haplotype*	GCA_905404305.1
Span (Mb)	360
Number of contigs	114
Contig N50 length (Mb)	6.3
Number of scaffolds	54
Scaffold N50 length (Mb)	12.5
Longest scaffold (Mb)	17.4
BUSCO * genome score	C:99.0%[S:98.6%,D:0.4%],F:0.2%, M:0.8%,n:5,286

*BUSCO scores based on the lepidoptera_odb10 BUSCO set using v5.1.2. C= complete [S= single copy, D=duplicated], F=fragmented, M=missing, n=number of orthologues in comparison. A full set of BUSCO scores is available at https://blobtoolkit.genomehubs.org/view/ilAntCard3.1/dataset/CAJQEZ01/busco.

**Figure 3.**
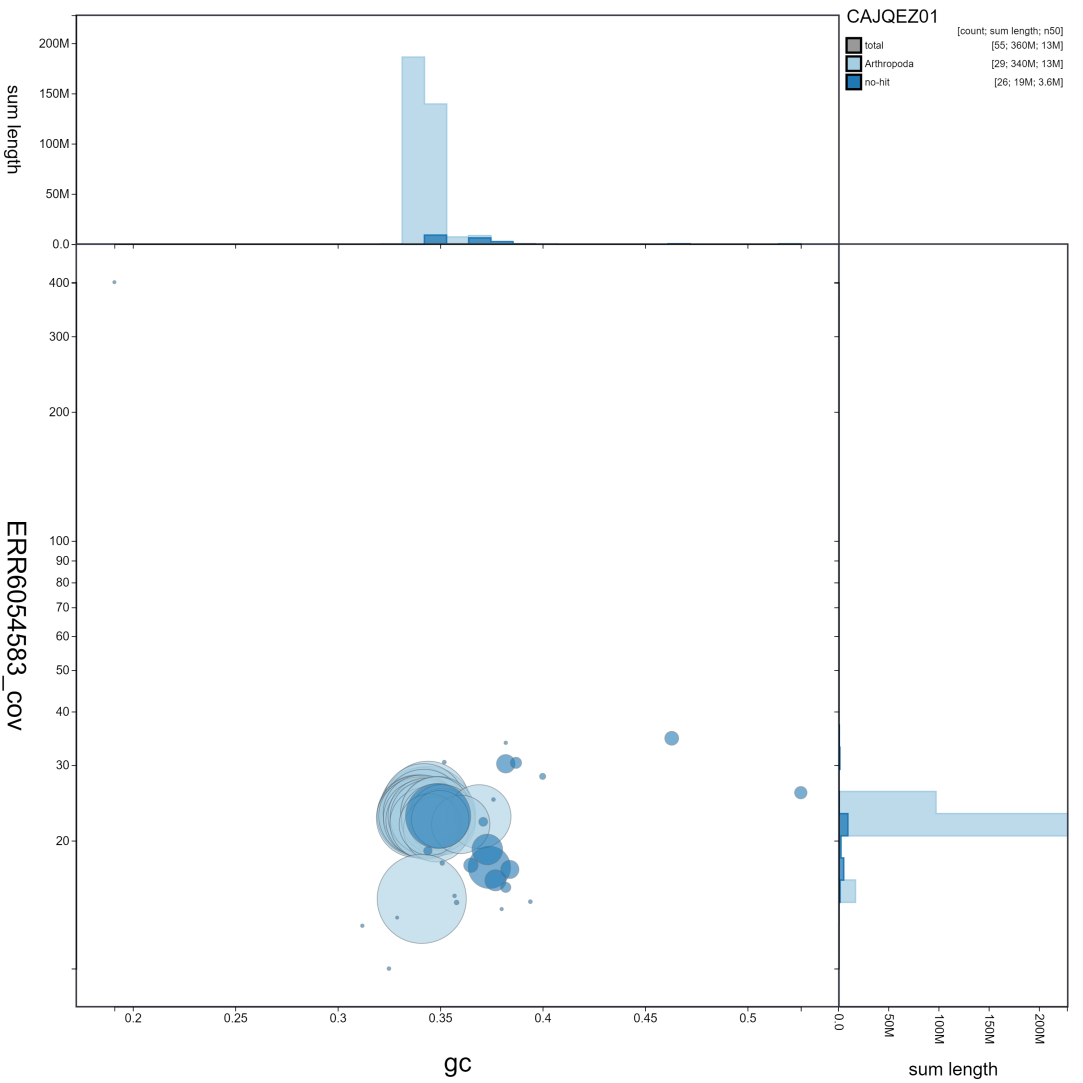
Genome assembly of *Anthocharis cardamines*, ilAntCard3.1: GC coverage. BlobToolKit GC-coverage plot. Scaffolds are coloured by phylum. Circles are sized in proportion to scaffold length. Histograms show the distribution of scaffold length sum along each axis. An interactive version of this figure is available at https://blobtoolkit.genomehubs.org/view/ilAntCard3.1/dataset/CAJQEZ01/blob.

**Figure 4.**
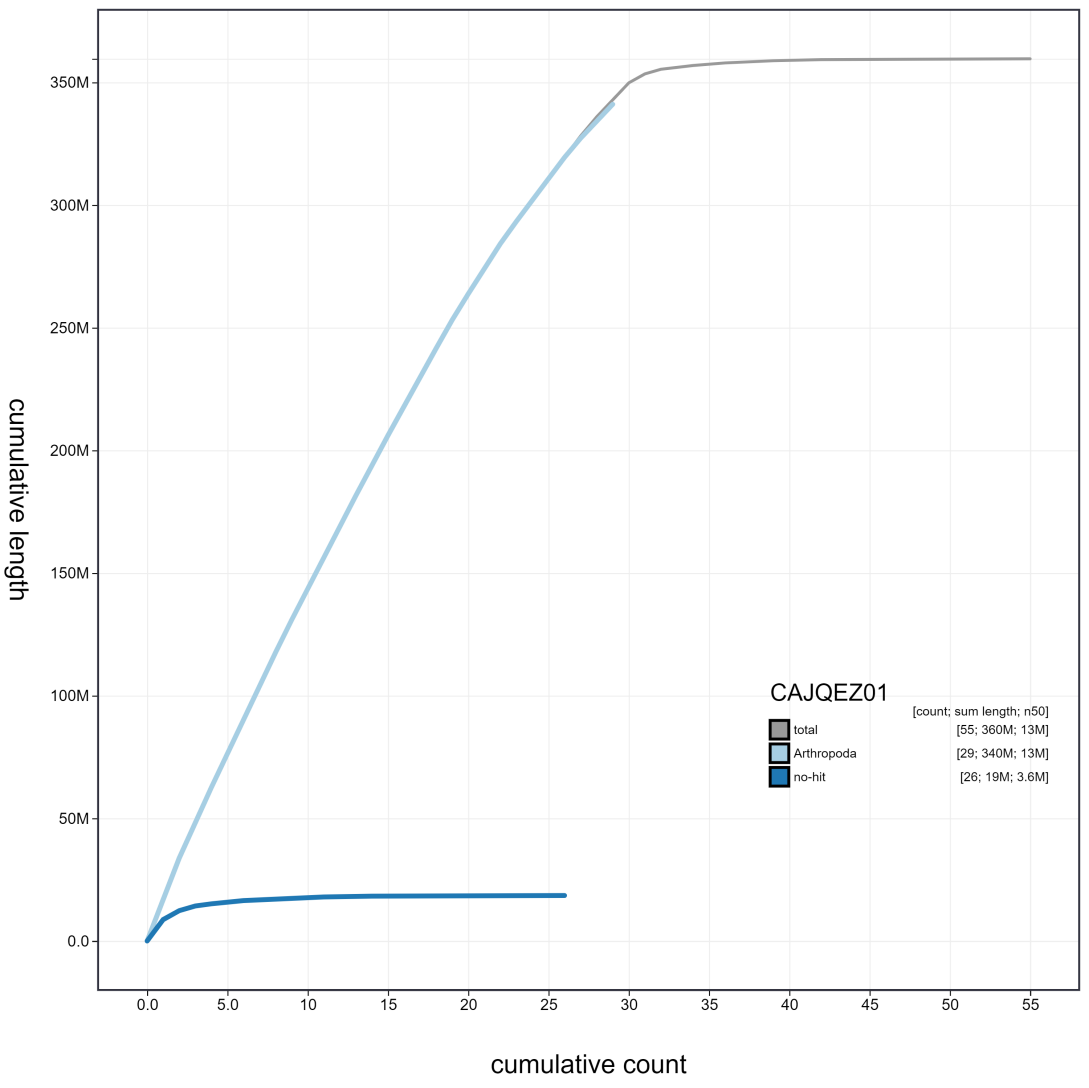
Genome assembly of *Anthocharis cardamines*, ilAntCard3.1: cumulative sequence. BlobToolKit cumulative sequence plot. The grey line shows cumulative length for all scaffolds. Coloured lines show cumulative lengths of scaffolds assigned to each phylum using the buscogenes taxrule. An interactive version of this figure is available at https://blobtoolkit.genomehubs.org/view/ilAntCard3.1/dataset/CAJQEZ01/cumulative.

**Figure 5.**
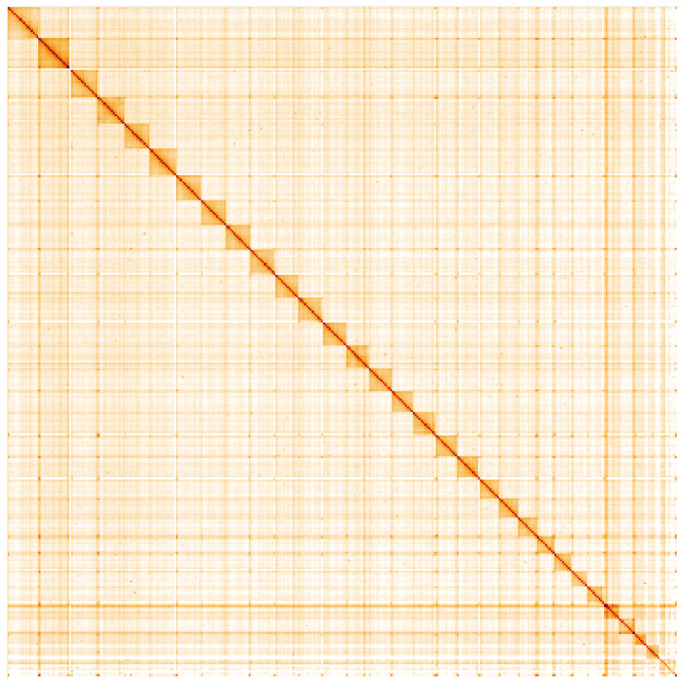
Genome assembly of *Anthocharis cardamines*, ilAntCard3.1: Hi-C contact map. Hi-C contact map of the ilAntCard3.1 assembly, visualised in HiGlass. Chromosomes are shown in size order from left to right and top to bottom. The interactive Hi-C map can be viewed at https://genome-note-higlass.tol.sanger.ac.uk/l/?d=ds_ts81KSRO_D0mSTg7guA.

**Table 2.** Chromosomal pseudomolecules in the genome assembly of *Anthocharis cardamines*, ilAntCard3.1.

INSDC accession	Chromosome	Size (Mb)	GC%
FR989950.1	1	17.37	34.4
FR989952.1	2	14.43	34.1
FR989953.1	3	14.38	34.4
FR989954.1	4	13.91	34.2
FR989955.1	5	13.89	34.2
FR989956.1	6	13.7	33.9
FR989957.1	7	13.48	33.9
FR989958.1	8	13.27	34.1
FR989959.1	9	12.76	34
FR989960.1	10	12.72	34.2
FR989961.1	11	12.7	33.8
FR989962.1	12	12.51	33.9
FR989963.1	13	12.35	34.1
FR989964.1	14	12.17	34.1
FR989965.1	15	11.88	34.2
FR989966.1	16	11.84	34.3
FR989967.1	17	11.78	34.4
FR989968.1	18	11.58	34.9
FR989969.1	19	10.66	34.3
FR989970.1	20	10.34	34.8
FR989971.1	21	10.07	34.8
FR989972.1	22	9.08	35.1
FR989973.1	23	8.97	35.1
FR989974.1	24	8.77	34.8
FR989975.1	25	8.73	34.9
FR989976.1	26	8.38	36.9
FR989977.1	27	7.81	34.5
FR989978.1	28	7.02	36
FR989979.1	29	6.82	35
FR989980.1	W	3.64	37.4
FR989951.1	Z	16.45	34.1
FR989981.1	MT	0.02	19.5
-	Unplaced	6.15	38.7

### Genome annotation report

The ilAntCard3.1 genome has been annotated using the Ensembl rapid annotation pipeline ( Table 1; https://rapid.ensembl.org/Anthocharis_cardamines_GCA_905404175.1/). The resulting annotation includes 28,207 transcribed mRNAs from 12,477 protein-coding and 4,279 non-coding genes. There are 1.82 coding transcripts per gene and 8.41 exons per transcript.

## Methods

### Sample acquisition and nucleic acid extraction

A single female *A. cardamines* specimen (ilAntCard3; genome assembly) and a single male *A. cardamines* specimen (ilAntCard2; HiC) were collected from Carrifran Wildwood, Scotland (latitude 55.4001, longitude -3.3352) using a net by Sam Ebdon, Gertjan Bisshop and Konrad Lohse (all University of Edinburgh). The samples were identified by Konrad Lohse and were snap-frozen at -80°C.

DNA was extracted at the Scientific Operations Core, Wellcome Sanger Institute. The ilAntCard3 sample was weighed and dissected on dry ice. Abdomen tissue was disrupted by manual grinding with a disposable pestle. Fragment size analysis of 0.01–0.5 ng of DNA was then performed using an Agilent FemtoPulse. High molecular weight (HMW) DNA was extracted using the Qiagen MagAttract HMW DNA extraction kit. Low molecular weight DNA was removed from a 200-ng aliquot of extracted DNA using 0.8X AMpure XP purification kit prior to 10X Chromium sequencing; a minimum of 50 ng DNA was submitted for 10X sequencing. HMW DNA was sheared into an average fragment size between 12–20 kb in a Megaruptor 3 system with speed setting 30. Sheared DNA was purified by solid-phase reversible immobilisation using AMPure PB beads with a 1.8X ratio of beads to sample to remove the shorter fragments and concentrate the DNA sample. The concentration of the sheared and purified DNA was assessed using a Nanodrop spectrophotometer and Qubit Fluorometer and Qubit dsDNA High Sensitivity Assay kit. Fragment size distribution was evaluated by running the sample on the FemtoPulse system.

### Sequencing

Pacific Biosciences HiFi circular consensus and 10X Genomics read cloud DNA sequencing libraries were constructed according to the manufacturers’ instructions. Sequencing was performed by the Scientific Operations core at the WSI on Pacific Biosciences SEQUEL II (HiFi) and Illumina HiSeq X (10X) instruments. Hi-C data were also generated from whole organism tissue of ilAntCard2 using the Qiagen Hi-C kit and sequenced on an Illumina HiSeq X (10X) instrument.

### Genome assembly

Assembly was carried out with HiCanu ( Nurk *et al.,* 2020)); haplotypic duplication was identified and removed with purge_dups ( Guan *et al.,* 2020). One round of polishing was performed by aligning 10X Genomics read data to the assembly with longranger align, calling variants with freebayes ( Garrison & Marth, 2012). The assembly was then scaffolded with Hi-C data ( Rao *et al.,* 2014) using SALSA2 ( Ghurye *et al.,* 2019). The assembly was checked for contamination and corrected using the gEVAL system ( Chow *et al.,* 2016) as described previously ( Howe *et al.,* 2021). Manual curation ( Howe *et al.,* 2021) was performed using gEVAL, HiGlass ( Kerpedjiev *et al.,* 2018) and Pretext. The mitochondrial genome was assembled using MitoHiFi ( Uliano-Silva *et al.,* 2021), which performed annotation using MitoFinder ( Allio *et al.,* 2020). The genome was analysed and BUSCO scores generated within the BlobToolKit environment ( Challis *et al.,* 2020). Table 3 contains a list of all software tool versions used, where appropriate.

**Table 3.** Software tools used.

Software tool	Version	Source
HiCanu	2.1	Nurk *et al.,* 2020
purge_dups	1.2.3	Guan *et al.,* 2020
SALSA2	2.2	Ghurye *et al.,* 2019
longranger align	2.2.2	https://support.10xgenomics.com/ genome-exome/software/pipelines/ latest/advanced/other-pipelines
freebayes	1.3.1-17- gaa2ace8	Garrison & Marth, 2012
MitoHiFi	1	Uliano-Silva *et al.,* 2021
gEVAL	N/A	Chow *et al.,* 2016
HiGlass	1.11.6	Kerpedjiev *et al.,* 2018
PretextView	0.1.x	https://github.com/wtsi-hpag/ PretextView
BlobToolKit	2.6.4	Challis *et al.,* 2020

### Gene annotation

The Ensembl gene annotation system ( Aken *et al.,* 2016) was used to generate annotation for the *Anthocharis cardamines* assembly ( GCA_905404175.1). Annotation was created primarily through alignment of transcriptomic data to the genome, with gap filling via protein-to-genome alignments of a select set of proteins from UniProt ( UniProt Consortium, 2019).

## Data Availability

European Nucleotide Archive: Anthocharis cardamines (orange tip). Accession number PRJEB43792; https://identifiers.org/ena.embl/PRJEB43792. The genome sequence is released openly for reuse. The *A. cardamines* genome sequencing initiative is part of the Darwin Tree of Life (DToL) project. All raw sequence data and the assembly have been deposited in INSDC databases. Raw data and assembly accession identifiers are reported in Table 1.

## Author information

Members of the Wellcome Sanger Institute Tree of Life programme are listed here: https://doi.org/10.5281/zenodo.6866293.

Members of Wellcome Sanger Institute Scientific Operations: DNA Pipelines collective are listed here: https://doi.org/10.5281/zenodo.5746904.

Members of the Tree of Life Core Informatics collective are listed here: https://doi.org/10.5281/zenodo.6125046.

Members of the Darwin Tree of Life Consortium are listed here: https://doi.org/10.5281/zenodo.6418363.
