# Supplementary material for: The genome sequence of the orange-tip butterfly, Anthocharis cardamines (Linnaeus, 1758)
Source: Wellcome Open Res. 2022 Oct 13;7:260. [Version 1] doi: 10.12688/wellcomeopenres.18117.1 (PMC9643846; doi:10.12688/wellcomeopenres.18117.1)
Supplement: Peer review history

# Peer review: Reviewer response for version 1

**DOI:** 10.21956/wellcomeopenres.20088.r52858

**Published:** 2022-11-08

**Authors:** Yu Okamura

**Affiliations:** 1 Department of Insect Symbiosis, Max Planck Institute for Chemical Ecology, Jena, Germany; 2 Department of Biological Sciences, Graduate School of Science, University of Tokyo, Tokyo, Japan

The article "The genome sequence of the orange-tip butterfly, Anthocharis cardamines (Linnaeus, 1758)" reported the first genome assembly for the orange-tip butterfly. The methods for assembly, scaffolding, and annotation are mostly well described and of a high standard. Also, the quality of the assembly is really high.

 I have only two concerns about the manuscript. First, the method for genome polishing with 10x data was not well explained. It is described up to the variant calling phase, but the actual way to polish the genome should be stated. Second, the annotation quality was not confirmed. Running a BUSCO on the annotation might be a help to see how well the genome was annotated.

 I would also like to ask the authors to add information about which subspecies are used for this study. According to the “Background” section, it seems it’s *britannica* but stating this clearly in the “Methods” section would help the readers to get important information about the actual samples used for this study.

Are sufficient details of methods and materials provided to allow replication by others?

Partly

Is the rationale for creating the dataset(s) clearly described?

Yes

Are the datasets clearly presented in a useable and accessible format?

Yes

Are the protocols appropriate and is the work technically sound?

Yes

Reviewer Expertise:

Insect genome assembly and annotation. Evolutionary Ecology. Butterfly genomics.

I confirm that I have read this submission and believe that I have an appropriate level of expertise to confirm that it is of an acceptable scientific standard.

# Peer review: Reviewer response for version 1

**DOI:** 10.21956/wellcomeopenres.20088.r52859

**Published:** 2022-11-07

**Authors:** Jason Hill

**Affiliations:** 1 Science for Life Laboratory, Department of Medical Biochemistry and Microbiology, Uppsala University, Uppsala, Sweden

In the article "The genome sequence of the orange-tip butterfly, *Anthocharis cardamines* (Linnaeus, 1758)" the authors Ebdon *et al.* report the genome assembly and annotation of an individual orange tip butterfly from Scotland.

 This effort appears to be an addition to the Darwin Tree of Life project and so it's sole focus on the generation of a high quality genome assembly is understandable. The methods used are generally considered the best available at present, and the resulting assembly and annotation conform to expectations. A brief exploration of the raw data revealed no anomalies to this reviewer.

Are sufficient details of methods and materials provided to allow replication by others?

Yes

Is the rationale for creating the dataset(s) clearly described?

Yes

Are the datasets clearly presented in a useable and accessible format?

Yes

Are the protocols appropriate and is the work technically sound?

Yes

Reviewer Expertise:

Genome assembly and annotation. Butterfly, fish, and bird genomics. Evolutionary genomics.

I confirm that I have read this submission and believe that I have an appropriate level of expertise to confirm that it is of an acceptable scientific standard.
